# Immunogenicity and safety of the CoronaVac vaccine in patients with cancer receiving active systemic therapy

**DOI:** 10.2217/fon-2021-0597

**Published:** 2021-08-03

**Authors:** Cengiz Karacin, Tulay Eren, Esra Zeynelgil, Goksen Inanc Imamoglu, Mustafa Altinbas, Ibrahim Karadag, Fatma Bugdayci Basal, Irem Bilgetekin, Osman Sutcuoglu, Ozan Yazici, Nuriye Ozdemir, Ahmet Ozet, Yesim Yildiz, Selin Akturk Esen, Gokhan Ucar, Dogan Uncu, Bedia Dinc, Musa Baris Aykan, İsmail Erturk, Nuri Karadurmus, Burak Civelek, İsmail Çelik, Yakup Ergun, Mutlu Dogan, Omur Berna Oksuzoglu

**Affiliations:** ^1^Department of Medical Oncology, Recep Tayyip Erdogan University Training & Research Hospital, Rize, Turkey; ^2^Department of Medical Oncology, HSU Dr Abdurrahman Yurtaslan Oncology Training & Research Hospital, Ankara, Turkey; ^3^Department of Medical Oncology, HSU Diskapi Yildirim Beyazit Training & Research Hospital, Ankara, Turkey; ^4^Department of Medical Oncology, Gazi University, Ankara, Turkey; ^5^Department of Infectious Diseases & Clinical Microbiology, Gazi University, Ankara, Turkey; ^6^Department of Medical Oncology, Turkish Ministry of Health Ankara City Hospital, Ankara, Turkey; ^7^Department of Medical Microbiology, Turkish Ministry of Health Ankara City Hospital, Ankara, Turkey; ^8^Department of Medical Oncology, HSU Gulhane Training & Research Hospital, Ankara, Turkey; ^9^Department of Medical Oncology, A Life Hospital, Ankara, Turkey; ^10^Department of Preventive Oncology, Institute of Oncology, Hacettepe University, Ankara, Turkey; ^11^Department of Medical Oncology, Batman Training & Research Hospital, Batman, Turkey

**Keywords:** cancer, chemotherapy, COVID-19, immunogenicity, immunotherapy, monoclonal antibody, safety, tumors, vaccine

## Abstract

**Aim:** To evaluate the immunogenicity and safety of the CoronaVac vaccine in patients with cancer receiving active systemic therapy. **Methods:** This multicenter, prospective, observational study was conducted with 47 patients receiving active systemic therapy for cancer. CoronaVac was administered as two doses (3 μg/day) on days 0 and 28. Antibody level higher than 1 IU/ml was defined as ‘immunogenicity.’ **Results:** The immunogenicity rate was 63.8% (30/47) in the entire patient group, 59.5% (25/42) in those receiving at least one cytotoxic drug and 100% (five of five) in those receiving monoclonal antibody or immunotherapy alone. Age was an independent predictive factor for immunogenicity (odds ratio: 0.830; p = 0.043). **Conclusion:** More than half of cancer patients receiving active systemic therapy developed immunogenicity.

## Tweetable abstract

Immunogenicity developed with CoronaVac in 25 (59.5%) of 42 patients who received at least one cytotoxic drug and in all patients (n = 5) who received monoclonal antibody or immunotherapy alone.

The coronavirus disease 2019 (COVID-19) pandemic has affected millions of people worldwide and caused more than 3 million deaths [1]. Advanced age and chronic disease are major risk factors for increased COVID-19 morbidity and mortality [2]. Cancer patients constitute a particular subgroup that needs more care because of delays in diagnostic and therapeutic processes during the pandemic leading to higher mortality rates [3,4]. Vaccines developed against COVID-19 have been promising for cancer patients as well as healthy individuals [5].

CoronaVac is an inactivated COVID-19 vaccine that has been shown to have immunogenicity, with vaccine-induced neutralizing antibodies to SARS coronavirus 2 (SARS-CoV-2) that can neutralize ten representative strains of SARS-CoV-2 [6,7]. In a phase II study, a highly automated bioreactor (ReadyToProcess WAVE 25 rocker; Cytiva, Umeå, Sweden) was used to produce the vaccine. Immunogenicity is provided by the high content of intact spike proteins in the vaccine. It has been used in many countries, including China and Turkey. The CoronaVac vaccine was approved by World Health Organization (WHO) after results of the phase III trial's interim analysis [8].

Experiences from influenza vaccine trials have given rise to thinking about possible lower immunogenicity rates in patients who are on active immunosuppressive therapy [9,10]. However, seasonal influenza vaccines have a protective effect even in cancer patients who receive active systemic treatment, although they develop less immunogenicity than healthy people [9]. In COVID-19 vaccine trials, receiving immunosuppressive therapy was an exclusion criterion, so patients on immunosuppressants (including cancer patients) were not included in the trials [6,7]. This therefore obscures the effectiveness of the COVID-19 vaccine in patients with a cancer diagnosis. Although there are no randomized controlled clinical trial data evaluating the immunogenicity of the COVID-19 vaccine in cancer patients who are on active systemic therapy, the COVID-19 vaccine is recommended for these patients by leading and local guidelines [11,12]. This multicenter, prospective, observational study aimed to evaluate the immunogenicity and safety of the CoronaVac vaccine in patients with solid organ tumors receiving active systemic therapy (cytotoxic chemotherapy, monoclonal antibody, immunotherapy).

## Methods

This multicenter, prospective, observational study was conducted with patients diagnosed with solid organ tumors receiving active systemic therapy. Ethics committee approval (2021-01/963) and Ministry of Health permission for the study were obtained on January 13, 2021. An informed consent form was obtained from all patients included in the study. Patients who had a solid organ tumor diagnosis, active systemic therapy (cytotoxic chemotherapy, monoclonal antibody, immunotherapy), Eastern Cooperative Oncology Group performance status 0–2, life expectancy >12 weeks, age >18 years and negative SARS-CoV-2 antibody serology before the first vaccine dose were included in the study. Those who had previous COVID-19 infection, contact with COVID-19-infected people in the last 14 days or any other immunosuppressive disease (i.e., HIV infection, solid organ transplant) were excluded from the study.

Evaluation of vaccine immunogenicity was the primary outcome of the study. Secondary outcomes were determining side effects, safety and factors affecting vaccine immunogenicity (e.g., age, sex, systemic treatment regimen). Baseline blood samples to measure SARS-CoV2 antibody level were taken 0–3 days before administration of the first dose of the vaccine. There was no intervention in planned systemic treatment schedules. A second dose of the vaccine was administered 4 weeks after the first dose. Side effects were recorded after the first and second doses. A second blood sample was taken to measure antibody level 4 weeks after the last dose of the vaccine. All patients were vaccinated within the Ministry of Health's vaccination program.

### Vaccine procedure

CoronaVac is an inactivated vaccine against COVID-19. The vaccine (3 μg in 0.5 ml of aluminum hydroxide diluent per dose in ready-to-use syringes) was administered intramuscularly according to a dosing schedule of day 0 and day 28. Since the study was noninterventional, a specific day was not determined between the patients' systemic treatment and administration of the vaccine by investigators. The median interval between the first dose of the vaccine and start of the previous chemotherapy cycle was 7 days (interquartile range: 5–10 days). The median interval between the second dose of the vaccine and start of the previous chemotherapy cycle was 7 days (interquartile range: 5–8 days).

### Interpretation of antibody results & assessment of immunogenicity

SARS-COV-2 antibody was evaluated by Siemens Healthcare Diagnostics (Tarrytown, NY, USA) Atellica IM SARS-CoV-2 total ELISA kits approved by the US FDA. The system reports Atellica IM SARS-CoV-2 total assay results in index values and as nonreactive (<1 index) or reactive (≥1.0 index) [13]. Seroconversion (immunogenicity) was defined as post-vaccination positivity of SARS-COV-2 antibody (≥1 IU) that was negative (<1 IU) before vaccination. The antibody meter ranged from 0.05 to 10 IU, and values higher than 10 IU were reported as >10 IU. According to serum antibody level, immunogenicity was classified as low (1–5 IU), intermediate (6–10 IU), or high (>10 IU).

### Statistical analysis

In the descriptive statistics of the study, numerical data were given as median (range or interquartile range) and categorical data as frequency (percentage). The Mann–Whitney U test was used to compare the continuous variables of the two independent groups. Pearson's chi-square or Fisher's exact test was used to compare categorical data. Variables with a p < 0.20 as a result of univariate analysis were included in the logistic regression analysis to determine the factors affecting immunogenicity. Statistical analysis was performed with SPSS Statistics 25.0 (IBM Corporation, NY, USA) for Windows (Microsoft Corporation, WA, USA), and a two-tailed p < 0.05 was considered statistically significant.

## Results

### Patient characteristics

A total of 47 patients with solid tumors were enrolled consecutively between 25 January 2021, and 26 April 2021. The median patient age was 73 years (range: 64–80), and 61.7% were male. Primary cancer sites, in order of frequency, were colorectal, breast, lung, genitourinary, gastric, pancreas, gynecological, biliary tract, and CNS. The majority of patients were diagnosed with stage IV disease and received palliative systemic treatment. There were 42 (89.4%) patients receiving at least one cytotoxic drug, three (6.4%) receiving monoclonal antibody alone and two (4.2%) receiving immunotherapy alone. Granulocyte colony-stimulating factor was administered to 36.2% of the patients (Tables 1 & 2).

**Table 1.** Demographic and clinical features of the patients.

Demographic and clinical features	Patients (n = 47)
Age (years), median (range)	73 (64–80)
Sex, n (%)	
Male	29 (61.7)
Female	18 (38.3)
Primary malignancy, n (%)	
Colorectal	13 (27.7)
Breast	7 (14.9)
Lung	6 (12.8)
Genitourinary	6 (12.8)
Gastric	5 (10.6)
Pancreas	4 (8.5)
Gynecological	3 (6.4)
Biliary tract	2 (4.2)
CNS	1 (2.1)
TNM stage, n (%)	
II	4 (8.5)
III	10 (21.3)
IV	33 (70.2)
Treatment modality, n (%)	
Neoadjuvant	1 (2.1)
Adjuvant	15 (31.9)
Palliative	31 (66.0)
Type of anticancer treatment, n (%)	
Receiving at least one cytotoxic drug	42 (89.4)
Receiving only monoclonal antibody	3 (6.4)
Receiving only immunotherapy	2 (4.2)
Treatment group, n (%)	
3W	10 (21.3)
2W	22 (46.8)
1W	7 (14.9)
C	6 (12.8)
IO	2 (4.2)
G-CSF, n (%)	
No	30 (63.8)
Yes	17 (36.2)

1W: Cytotoxic drug or monoclonal antibody given each week; 2W: Cytotoxic drug or monoclonal antibody given every 2 weeks; 3W: Cytotoxic drug or monoclonal antibody given every 3 weeks; C: Cytotoxic drug given continuously orally; G-CSF: Granulocyte colony-stimulating factor; IO: Immunotherapy given every 2 weeks; TNM: Tumor, node, metastasis.

**Table 2.** Details of patient demographics, clinical features, treatment schedules and immunogenicity results.

Group	Age (years)	Sex	ECOG PS	Comorbidity	Primary	Stage	Regimen	G-CSF	Antibody IU/ml	Seroconversion
3W	64	F	1	DM, HT	Breast	III	Trastuzumab	N	>10	Y
3W	72	F	1	HT	Breast	IV	Trastuzumab	N	>10	Y
3W	74	F	0	DM, HT	Breast	III	Doxorubicin + cyclophosphamide	N	6.82	Y
3W	65	F	1	DM, HT	Breast	IV	Pertuzumab + trastuzumab	N	>10	Y
3W	65	F	1	HT, COPD	Lung	II	Etoposide + cisplatin	N	2.87	Y
3W	70	M	2	CHF	Lung	IV	Paclitaxel + carboplatin	N	>10	Y
3W	75	M	2	–	Lung	III	Paclitaxel + carboplatin	Y	0.27	N
3W	74	M	0	–	Prostate	IV	Docataxel	Y	0.87	N
3W	74	M	1	HT, CAD	Prostate	IV	Docetaxel	Y	0.64	N
3W	74	M	1	–	Gastric	IV	Docetaxel + cisplatin + 5-FU	Y	0.59	N
2W	80	M	1	–	Gastric	IV	FOLFIRI	Y	1.12	Y
2W	71	M	0	HT, CAD	Colon	IV	FOLFIRI + cetuximab	N	6.82	Y
2W	75	F	1	HT, DM	GBM	IV	Irinotecan + bevacizumab	N	>10	Y
2W	80	F	1	HT	Bladder	IV	Paclitaxel + carboplatin	Y	0.90	N
2W	73	M	1	DM	Colon	IV	FUFA + bevacizumab	N	1.58	Y
2W	69	M	0	–	Pancreas	IV	Gemcitabine 1–8	N	0.98	N
2W	80	F	1	HT	Colon	IV	FUFA + bevacizumab	N	5.29	Y
2W	71	M	1	DM, HT, COPD	Pancreas	IV	mFOLFIRINOX	Y	1.20	Y
2W	73	F	1	HT	Colon	IV	FOLFIRI	N	2.78	Y
2W	71	M	1	HT, DM	Colon	III	FUFA	N	6.31	Y
2W	72	M	1	Arrhythmia	Colon	IV	FOLFIRI + cetuximab	Y	9.15	Y
2W	78	M	1	Asthma	Pancreas	III	Gemcitabine	Y	1.66	Y
2W	74	M	1	HT, COPD	Gastric	III	FUFA	N	4.86	Y
2W	75	M	1	CAD	Colon	IV	FOLFIRI	Y	0.76	N
2W	72	F	1	–	Breast	IV	Gemcitabine	Y	0.98	Y
2W	72	M	0	HT	Bladder	IV	Gemcitabine + carboplatin	N	2.66	Y
2W	78	F	2	HT, DM	Endometrium	IV	Paclitaxel + carboplatin	N	0.86	N
2W	77	F	1	HT, COPD	Ovarian	IV	Gemcitabine	Y	0.05	N
2W	68	M	1	HT	Gastric	III	FLOT4	Y	1.05	Y
2W	65	M	1	HT, CAH	Rectum	IV	FOLFOX	N	4.42	Y
2W	77	F	2	HT, DM	Pancreas	IV	FOLFIRI	Y	>10	Y
2W	76	M	1	HT, DM, CAD	Biliary tract	IV	Gemcitabine + cisplatin	N	0.83	N
1W	73	M	1	–	Lung	IV	Paclitaxel	Y	1.05	Y
1W	77	M	1	CAH	Lung	IV	Irinotecan	N	0.19	N
1W	80	F	1	HT, DM, arrhythmia	Breast	III	Paclitaxel	Y	0.45	N
1W	66	F	0	–	Breast	II	Paclitaxel	N	0.97	N
1W	67	M	0	–	Rectum	IV	5-FU	N	>10	Y
1W	77	F	1	HT	Ovarian	IV	Paclitaxel + carboplatin	Y	7.20	Y
1W	70	M	0	–	Lung	III	Carboplatin	N	1.07	Y
C	73	F	1	–	Biliary tract	IV	Capecitabine	N	1.03	Y
C	73	M	1	Asthma	Colon	II	Capecitabine	N	1.59	Y
C	72	M	1	DM	Colon	II	XELOX	N	4.42	Y
C	73	M	2	–	Gastric	III	XELOX	N	0.80	Y
C	71	F	2	HT, DM	Rectum	IV	Capecitabine + cetuximab	N	0.05	N
C	76	M	1	–	Colon	IV	Capecitabine	N	0.95	N
IO	71	M	0	–	RCC	IV	Nivolumab	N	2.06	Y
IO	76	M	1	–	RCC	IV	Nivolumab	N	1.93	Y

1W: Cytotoxic drug or monoclonal antibody given each week; 2W: Cytotoxic drug or monoclonal antibody given every 2 weeks; 3W: Cytotoxic drug or monoclonal antibody given every 3 weeks; 5-FU: Fluorouracil; C: Cytotoxic drug given continuously orally; CAD: Coronary artery disease; CAH: Congenital adrenal hyperplasia; CHF: Congestive heart failure; COPD: Chronic obstructive pulmonary disease; DM: Diabetes mellitus; ECOG PS: Eastern Cooperative Oncology Group performance status; F: Female; FOLFIRI: Folinic acid, fluorouracil and irinotecan; FOLFOX: Folinic acid, fluorouracil and oxaliplatin; FUFA: Fluorouracil and folinic acid; FLOT4: fluorouracil plus leucovorin, oxaliplatin, and docetaxel; GBM: Glioblastoma multiforme; G-CSF: Granulocyte colony-stimulating factor; HT: Hypertension; IO: Immunotherapy given every 2 weeks; M: Male; mFOLFIRINOX: Modified folinic acid, fluorouracil, irinotecan and oxaliplatin; N: No; RCC: Renal cell carcinoma; TNM: Tumor, node, metastasis; XELOX: Capecitabine and oxaliplatin; Y: Yes.

### Immunogenicity

Of the 47 patients, 30 (63.8%) had seroconversion (immunogenicity). Immunogenicity developed in all five patients who received monoclonal antibody (n = 3) or immunotherapy (n = 2) alone. Immunogenicity also developed in 25 (59.5%) of 42 patients who received at least one cytotoxic drug. Antibody levels in all patients who received monoclonal antibodies were found to be higher (>10 IU) and were slightly elevated (1–5 IU) in two patients who received immunotherapy alone. Of the 25 patients who received at least one systemic cytotoxic treatment and developed immunogenicity, high (>10 IU) antibody levels were measured in four, moderate (6–10 IU) levels were measured in six and low (1–5 IU) levels were measured in 15. Detailed patient demographics, clinical characteristics and antibody levels are shown in Table 3.

**Table 3.** Univariate analysis of serological response rate.

	Seroconversion		p-value
	No	Yes	
Age (years), median (IQR)	75 (73–77)	72 (70–74)	0.031
Sex, n (%)			
Male	10 (34.5)	19 (65.5)	0.760
Female	7 (38.9)	11 (61.1)	
ECOG PS, n (%)			
0	3 (33.3)	6 (66.7)	0.249
1	10 (31.3)	22 (68.8)	
2	4 (66.7)	2 (33.3)	
Comorbidity, n (%)			
No	7 (50.0)	7 (50.0)	0.199
Yes	10 (30.3)	23 (69.7)	
TNM stage, n (%)			
II	1 (25.0)	3 (75.0)	0.767
III	3 (30.0)	7 (70.0)	
IV	13 (39.4)	20 (60.6)	
Treatment, n (%)			
Palliative	13 (41.9)	13 (58.1)	0.252
Other	4 (25.0)	12 (75.0)	
Treatment group, n (%)			
1W	3 (42.9)	4 (57.1)	NA
2W	7 (31.8)	15 (68.2)	
3W	4 (57.1)	3 (42.9)	
C	3 (50.0)	3 (50.0)	
IO	0 (0)	2 (100)	
Monoclonal AB only	0 (0)	3 (100)	
G-CSF, n (%)			
No	8 (26.7)	22 (73.3)	0.072
Yes	9 (52.9)	8 (47.1)	

1W: Cytotoxic drug or monoclonal antibody given each week; 2W: Cytotoxic drug or monoclonal antibody given every 2 weeks; 3W: Cytotoxic drug or monoclonal antibody given every 3 weeks; AB: Antibody; C: Cytotoxic drug given continuously orally; ECOG PS: Eastern Cooperative Oncology Group performance status; G-CSF: Granulocyte colony-stimulating factor; IO: Immunotherapy given every 2 weeks; IQR: Interquartile range; NA: Not applicable; TNM: Tumor, node, metastasis.

In univariate analysis, patients who had immunogenicity were younger, with a median age of 72 years (p = 0.031), whereas the median age of those who had no seroconversion was 75 years. The immunogenicity rate was lower in those who used granulocyte colony-stimulating factor (47.1% vs. 73.3%; p = 0.072). There was no relationship between immunogenicity and other demographic and clinical characteristics (Table 3).

Age was defined as a significant independent predictive factor for CoronaVac immunogenicity in multivariate analysis (odds ratio: 0.830; 95% CI: 0.693–0.994; p = 0.043) (Table 4). None of the patients had COVID-19 infection at a median follow-up of 85 days (range: 62–98 days).

**Table 4.** Multivariate analysis of serological response.

	OR	95% CI	p-value
Comorbidity	2.937	0.729–11.833	0.130
G-CSF	0.468	0.116–1.881	0.284
Age	0.830	0.693–0.994	0.043

G-CSF: Granulocyte colony-stimulating factor; OR: Odds ratio.

### Safety analysis

Local and systemic reactions after the first and second doses of the vaccine are shown in Table 5. After the first and second doses, side effect rates of any grade were 18.9 and 23.1%, respectively. With regard to local reactions, pain at the injection site was the most common side effect; among systemic side effects, fatigue was the most common. There were no serious (grade 3 or 4) side effects or toxic deaths.

**Table 5.** Local and systemic reactions after first and second vaccine doses.

	First dose			Second dose		
	Any grade	Grade 1	Grade 2	Any grade	Grade 1	Grade 2
Total, n (%)	9 (18.9)	7 (14.7)	2 (4.2)	11 (23.1)	8 (16.8)	3 (6.3)
Local reaction, n (%)						
Pain at injection site	2 (4.2)	2 (4.2)	0 (0)	3 (6.3)	3 (6.3)	0 (0)
Swelling	1 (2.1)	1 (2.1)	0 (0)	0 (0)	0 (0)	0 (0)
Itchiness	1 (2.1)	1 (2.1)	0 (0)	0 (0)	0 (0)	0 (0)
Erythema	0 (0)	0 (0)	0 (0)	2 (4.2)	0 (0)	2 (4.2)
Systemic reaction, n (%)						
Fever	1 (2.1)	1 (2.1)	0 (0)	1 (2.1)	1 (2.1)	0 (0)
Myalgia	1 (2.1)	1 (2.1)	0 (0)	0 (0)	0 (0)	0 (0)
Fatigue	2 (4.2)	0	2 (4.2)	5 (10.5)	4 (8.4)	1 (2.1)
Headache	1 (2.1)	1 (2.1)	0 (0)	0 (0)	0 (0)	0 (0)

## Discussion

In this study, the authors prospectively evaluated the immunogenicity and safety of the CoronaVac vaccine in patients with solid organ tumors receiving active systemic therapy. The immunogenicity rate was 63.8% for the whole patient population and 59.5% for the patients who received at least one cytotoxic chemotherapy. The phase I and II CoronaVac trial, which evaluated the immunogenicity of the CoronaVac vaccine in healthy 18- to 59-year-old individuals, had four cohorts, and 3 and 6 μg of the vaccine was administered on a schedule of 0–14 and 0–28 days [6]. However, in the authors' study, the vaccine was administered on days 0 and 28 at a dose of 3 μg. In the phase I and II CoronaVac trial, the immunogenicity rates were 95.0 and 96.5% for doses of 3 and 6 μg (days 0 and 28), respectively. Another phase I and II trial evaluated the immunogenicity and safety of the CoronaVac vaccine in a healthy elderly population (≥60 years) [7], and the immunogenicity rates were 98.0 and 99.0% in the 3 and 6-μg dose subgroups, respectively. In the present study, the immunogenicity rates with 3 μg (days 0 and 28) were lower than those seen in these phase I and II CoronaVac trials. However, this study included cancer patients who were undergoing active systemic cancer treatment with chemotherapy, monoclonal antibody or immunotherapy. Although the immunogenicity rate was relatively lower in cancer patients, none had COVID-19 over a median follow-up period of 85 days.

To the authors' knowledge, this is the first study to evaluate the immunogenicity of the CoronaVac vaccine in cancer patients receiving active systemic therapy. The low immunogenicity demonstrated in the authors' study was consistent with other studies [14–17]. In a study conducted in Turkey, it was shown that patients using immunomodulators for rheumatological disease developed less immunogenicity compared with healthy individuals receiving the CoronaVac vaccine [14]. Similar results have been found in cancer patients who received the mRNA-1273 (Moderna, MA, USA) or BNT162b2 mRNA (Pfizer, NY, USA) COVID-19 vaccines [15–17]. The immunogenicity rate was found to be 53.7% in patients with hematological malignancies, of which approximately 45% received active systemic therapy [15]. In the same study, it was stated that immunogenicity decreased independently of treatment in patients with chronic lymphocytic leukemia. In another study evaluating 167 patients with chronic lymphocytic leukemia, the immunogenicity rate was found to be 39.5% with the BNT162b2 mRNA COVID-19 vaccine [16]. In a study by Massarweh *et al.* that included patients with solid organ tumors or hematological malignancies receiving active systemic therapy, it was shown that the mean antibody level detected after vaccination (BNT162b2 mRNA) was lower than that seen in healthy individuals [17].

In previous influenza vaccine studies, it has been shown that the immunogenicity rate may be lower in immunosuppressive patients compared with healthy individuals [9]. Adjuvant and high-dose vaccines are beneficial for increasing immunogenicity in seasonal influenza vaccines in immunosuppressive patients. It was also shown in a meta-analysis that the immunogenicity of the influenza vaccine was lower in cancer patients, who constituted the immunosuppressive group, compared with healthy individuals [9,18]. In the VACANSE study in which the immunogenicity of the H1N1v vaccine was evaluated in patients with solid organ tumors receiving active systemic treatment, it was reported that a single dose of the vaccine did not provide sufficient immunogenicity [10]. However, the immunogenicity might have increased had the vaccine been administered in two doses. Similarly, the fact that immunogenicity was lower in the authors' study compared with studies using healthy individuals raised the question of whether administration of a booster CoronaVac vaccine dose may increase the immunogenicity rates; this needs further clinical trials.

With aging, many molecular changes – called immunosenescence – occur in the immune system [19]. This dysregulation in the elderly immune system causes a decrease in the immune response obtained with vaccines. Considering that advanced age is a significant risk factor for COVID-19 morbidity and mortality, elderly patients have been given priority for vaccination against COVID-19 in many countries, including the authors' [20]. One of the concerns in the vaccination of elderly patients is immunogenicity sufficiency. The CoronaVac phase I and II trial, which was conducted with elderly volunteers, showed that the vaccine developed an immunogenicity profile comparable to that seen with young adults, without any serious adverse events [7]. The authors' study showed that the only independent factor affecting immunogenicity in multivariate analysis was age (p = 0.043). As mentioned, immunogenicity decreases with increasing age. This point might have also contributed to the lower immunogenicity rate seen with the CoronaVac vaccine in the authors' elderly cancer patients on active cancer treatment.

In the authors' study, the cumulative rate of possible vaccine-related side effects observed after two doses of the CoronaVac vaccine was 32%. Toxicity rates were reported to be 33 and 20% in the 3-μg cohorts of the Phase I and II CoronaVac trials, which were conducted with younger and elderly healthy volunteers, respectively [6,7]. The fatigue rate in the authors' study was higher than that seen in other CoronaVac trials (14.7 vs <10 and 3%). The higher fatigue rate in the authors' patients might have been related to cancer diagnosis and its active treatment during vaccination. Similar to the CoronaVac Phase I and II trials, no serious vaccine-related adverse events were observed in the authors' study.

Some researchers have hypothesized that the vaccine could hypothetically lead to an exaggerated immune response in immunotherapy recipients [21]. However, in a study evaluating short-term safety in 134 patients who received immunotherapy and the BNT162b2 mRNA COVID-19 vaccine, it was reported that there was no increase in immunotherapy-related immune side effects [22]. In the authors' study, only two patients received imunotherapy, and they did not experience any side effects. The median interval between the vaccine and the start of the previous immunotherapy cycle was 7 days in both patients.

This study did not have a validation cohort, which was a strong limitation. The study population also consisted of elderly patients, which was another limitation. Lower immunogenicity rate in the geriatric population irrespective of vaccination is a well-known finding, so it should be kept in mind that the study results do not reflect immunogenicity with vaccination in young cancer patients receiving active systemic therapy. It is a fact that the development of immunogenicity alone does not mean absolute protection from COVID-19 infection. Despite a median follow-up period of 85 days, the authors note that this is not long enough to comment on whether the vaccine has a long-term protective effect against COVID-19 infection. Another limitation was that cellular immunity, which has a preventive effect against COVID-19 infection, was not evaluated in this study. Comorbidities and active cancer treatment modalities might be confounding factors in the evaluation of ‘real’ vaccine-related side effects. Therefore, it has been stated that the side effects were ‘probably’ related to the vaccine. The low number of patients and absence of a control group are another limitation of the study. Despite these limitations, to the best of the authors' knowledge, this study was the first to evaluate the efficacy and safety of the CoronaVac vaccine in cancer patients undergoing active systemic cancer treatment with chemotherapy, monoclonal antibody or immunotherapy.

## Conclusion

Immunogenicity developed with two doses of the CoronaVac vaccine (3 μg/day days 0 and 28) in more than half of the patients with solid organ tumors undergoing active systemic cytotoxic chemotherapy.

## Future Perspective

The fact that vaccination rates do not reach the targeted levels worldwide and virus mutations show that our fight against COVID-19 will continue in the coming years. There is a need for studies investigating more effective vaccination programs in cancer patients receiving active systemic therapy.

**Summary points**

- This prospective observational multicenter study was conducted with 47 patients with solid organ tumors receiving active systemic therapy to evaluate the immunogenicity and safety of the CoronaVac vaccine in patients with solid organ tumors receiving active systemic therapy (cytotoxic chemotherapy, monoclonal antibody, immunotherapy).
- Evaluation of vaccine immunogenicity was the primary outcome of the study; the secondary outcome was determining the vaccine's safety.
- The median patient age was 73 (range: 64–80), and 61.7% were male. Immunogenicity developed in 25 (59.5%) of 42 patients who received at least one cytotoxic drug and in all patients (n = 5) who received monoclonal antibody or immunotherapy alone.
- In univariate analysis, patients who had immunogenicity were younger, with a median age of 72 years (p = 0.031), whereas the median age of those who had no seroconversion was 75 years.
- Immunogenicity developed in 47.1% of those who were administered granulocyte colony-stimulating factor and 73.3% of those who were not administered granulocyte colony-stimulating factor (p = 0.072).
- In multivariate analysis, the only independent predictive factor affecting immunogenicity was patient age (odds ratio: 0.830; 95% CI: 0.693–0.994; p = 0.043).
- After the first and second doses of the vaccine, side effect rates of any grade were 18.9 and 23.1%, respectively, and there were no serious (grade 3 or 4) side effects or toxic deaths.
- Immunogenicity developed with two doses of the CoronaVac vaccine (3 μg/day days 0 and 28) in more than half of the patients with solid organ tumors undergoing active systemic cytotoxic chemotherapy.
